# Physicians’ Experiences and Perceptions of Environmental Factors Affecting Their Practices of Continuous Deep Sedation until Death: A Secondary Qualitative Analysis of an Interview Study

**DOI:** 10.3390/ijerph19095472

**Published:** 2022-04-30

**Authors:** Stijn Vissers, Sigrid Dierickx, Lenzo Robijn, Joachim Cohen, Luc Deliens, Freddy Mortier, Kenneth Chambaere

**Affiliations:** 1 End-of-Life Care Research Group, Vrije Universiteit Brussel (VUB), 1090 Brussels, Belgium; sigrid.dierickx@ugent.be (S.D.); joachim.cohen@vub.be (J.C.); luc.deliens@vub.be (L.D.); freddy.mortier@ugent.be (F.M.); kenneth.chambaere@ugent.be (K.C.); 2 End-of-Life Care Research Group, Ghent University, 9000 Ghent, Belgium; 3 Department of Public Health and Primary Care, Ghent University, 9000 Ghent, Belgium; lenzo.robijn@ugent.be; 4 Bioethics Institute Ghent, Ghent University, 9000 Ghent, Belgium

**Keywords:** physicians, continuous deep sedation until death, environmental factors, palliative sedation, Belgium, culture, structure

## Abstract

As previous research has paid little attention to environmental factors affecting the practice of continuous deep sedation until death (CDS), we aimed to explore these using physicians’ experiences and perceptions. We performed an interpretative thematic analysis of primary data from a qualitative interview study conducted from February to May 2019 in Belgium with 47 physicians. Structural factors were identified: the lack of professional and/or technical support in monitoring sedated patients; the use of guidelines in team contexts; the time constraints for treating individual patients and work pressure; the structural knowledge gap in medical education; the legal context for assisted dying; and the lack of a clear legal context for CDS. Cultural factors were identified: the moral reservations of care teams and/or institutions towards CDS; the presence of a palliative care culture within care teams and institutions; the culture of fear of making clinical errors regarding CDS among a group of physicians; the professional stigma of performing assisted dying among some of the physician population; the different understandings of CDS in medical and policy fields; and the societal taboo around suffering at the end of life and natural death. To conclude, improving CDS practice requires a whole-system approach considering environmental factors.

## 1. Introduction

At the end of life, some patients might experience unbearable suffering from refractory symptoms that are extremely difficult or impossible to manage [1]. For these patients, continuous deep sedation until death (CDS) can be performed as an option of last resort to mitigate the suffering [2,3]. This involves the intentional lowering of patients’ consciousness in a deep and prolonged state until death, so that patients are no longer aware of their unbearable suffering [2].

While studies have shown that the prevalence of CDS is considerable [4], they have simultaneously established that it varies between countries [5]. A comparative cross-country study, for instance, found that prevalence differs between European countries, ranging from 2.1% of all deaths in Denmark to 8.5% in Italy [6]. It is notable that the authors of the study partially attributed the higher prevalence in Italy and Belgium to the countries’ overarching cultural–religious context. This reflects the claim of Anquinet et al., who connected the differences found in the prevalence of CDS between Belgium, the Netherlands, and the United Kingdom in part to legal, cultural, and organizational factors in these countries [7]. Secondly, the prevalence of CDS also varies substantially between care settings. In this respect, a systematic review demonstrated that the prevalence of CDS differs between hospitals, hospital departments, (palliative) care units, nursing homes, hospices, and home settings [5].

The practice of making decisions about and performing CDS, hereafter referred to as (CDS) practice, has also been found to differ between countries [5]. The international UNBIASED Study, for example, illustrates that rapid induction of CDS is more common in the Netherlands and Belgium, while proportional induction of CDS is the norm in the United Kingdom [8,9,10,11]. The authors of the study concluded that CDS encompasses a spectrum of practices that are partly embedded in national cultures, such as the legal and ethical context. Simultaneously, variations in CDS practice between care settings have been identified. Arevalo et al., for example, demonstrated that Dutch physicians were less often present at the start of CDS in nursing homes/hospices and hospitals than in CDS at home [12].

To understand the variations in CDS, the literature has particularly focused on the underlying factors affecting its practice. In that regard, clinical and (inter)personal factors in particular have been extensively examined [13]. (Inter)personal factors are non-clinical influences within the micro-context related to individuals and their interactions [14]. Among clinical factors, studies show an increased probability of patients with cancer, dyspnea, and psycho-existential suffering receiving CDS [13,15,16,17]. Among inter(personal) factors, studies demonstrate an increased probability of male patients, younger patients, and patients treated by physicians who are deeply secular and/or in favor of assisted dying receiving CDS [5,18].

While several authors have explicitly suggested that the environmental context might play a role in the variation of CDS [5,6,7,8,11,12], studies have rarely examined environmental factors affecting CDS practice. These factors, also called the ‘environmental context’, are influences within the meso and macro context that are external to individuals [14]. Insight into environmental factors might aid in addressing challenges that affect optimal practice of CDS. For example, some practitioners deploy CDS to hasten death in order to mitigate proxies’ burden [19]; suboptimal medication is sometimes used to initiate CDS [20]; proxies are often not involved in the decision making about CDS, and they may put practitioners under pressure to induce CDS [9,21]; and practitioners may experience emotional and moral distress throughout CDS practice [9]. Moreover, few quality-improvement initiatives have been successful in addressing these challenges in practice [22], which may indicate poor understanding and underestimation of the importance of the broader context. 

This article aims to explore physicians’ experiences and perceptions of the environmental factors affecting their CDS practices. The main research question of this study is: What are physicians’ experiences and perceptions of what environmental factors affect their practice of CDS? To that end, we conducted a secondary analysis on primary data from a qualitative interview study with Belgian physicians [23]. The particular focus on perceptions and experiences of physicians has been chosen because they are prominent protagonists in CDS practice. Moreover, they are often the only medical professionals who are legally authorized to perform CDS in most jurisdictions [24]. 

## 2. Methods

### 2.1. Study Design 

We performed a secondary study drawing on primary pre-existing data, namely in-depth interviews with physicians, from a broader qualitative primary study that explored physicians’ framing of CDS practice and its appropriate control measures [23]. Findings on the latter are reported elsewhere [23]. Following Heaton’s classification of secondary studies, our study design reflects an inside supplementary approach [25,26]. This involves the same researchers conducting an in-depth examination of an issue or aspect of the primary data that was not addressed or was only partly covered in the primary study [26,27]. Moreover, the emphasis might be on a particular issue or theme that emerged in the primary study [26,28]. In the first study, a subsidiary finding was that physicians in our sample pointed out several environmental factors impacting their CDS practices, especially when prompted to discuss the context in which their own CDS practices were embedded. Consequently, we decided to conduct a secondary study to explore these environmental factors in depth, thereby addressing the knowledge gap on this topic. Furthermore, performing a secondary study is deemed appropriate when the research aim is closely linked to the one of the primary study [29,30,31]. By conducting a secondary study, on the one hand, we endorse the methodological argument that re-analyzing primary data maximizes their fullest use [26]. On the other hand, we argue that this is particularly relevant as physicians are considered to be a difficult population to be recruited for qualitative research [30]. 

The Consolidated Criteria for Reporting Qualitative Research (COREQ) guidelines were followed to ensure rigor in the reporting of this article [32]. We discuss the methods of our primary and secondary studies in detail in the following sections.

### 2.2. Study Context

With regard to the study setting, we conducted the primary study in Belgium among physicians with experience in performing CDS. CDS is estimated to be performed prior to one out of every eight deaths in Belgium and to be more prevalent in hospital settings than in home settings [33,34]. Moreover, CDS practice in Belgium is currently not regulated by a specific legal framework stipulating its legal requirements [24]. However, the common legal perspective in Belgium categorizes CDS as a form of symptom control, thus being a ‘normal medical practice’ that physicians are legally allowed to employ [35]. This is also emphasized by various organizations and associations: for example, in the assisted dying reports of the Belgian Federal Control Commission for Euthanasia [36], in the guidelines for palliative sedation of The Federation of Palliative Care Flanders [37], in the ethical advice on palliative sedation from the Flemish network of care organizations Zorgnet–Icuro [38], and in the support guidelines for physicians of the Life End Information Forum [39]. 

### 2.3. Primary Study: Research Paradigm 

The research paradigm of our primary study, i.e., the set of beliefs and assumptions that guide the research process [40], was based on social constructionism [41]. As such, we sought to grasp the social phenomena of CDS practice, namely, the multiple understandings and diverse realities of how physicians define and experience the specific context of the practice and potential control measures [23]. 

### 2.4. Primary Study: Participants and Recruitment

Physicians with experience performing CDS were recruited. More specifically, eligibility criteria were: (a) having carried out CDS at least three times in the five years prior to the interview, including once during the past year; (b) residency in Belgium; (c) fluency in French and/or Dutch; (d) having given informed consent to participate in the study. Recruitment took place from January to March 2019 in Belgium.

To cover diverse experiences, both purposive and snowball sampling were deployed during participant recruitment, using a multistage strategy to maximize validity [42]. With regard to purposive sampling, an invitation letter explaining the eligibility criteria and study purpose was sent to (a) 40 hospitals (four hospitals in each Belgian province) and 30 general practices (three practices in each Belgian province), both groups identified through the website of the Belgian National Institute for Health and Disability Insurance; and to (b) national and regional physician and palliative care organizations. With regard to snowball sampling, participants were asked to identify other potential participants. We aimed to include a wide variety of participants in terms of age, sex, and regional location of professional practice. 

### 2.5. Primary Study: Data Collection

Authors S.V. and L.R. and a data manager conducted the interviews in the primary study between February and May 2019. Both authors have a background in health sociology, and the data manager has a background in nursing. All interviewers were familiar with interviewing on health-related or end-of-life care topics. We used a semi-structured interview guide containing open-ended questions about physicians’ experiences with CDS practice. More specifically, questions were centered around three main themes: (a) personal practices of CDS, (b) feasible control measures for CDS, and (c) solutions and actions for improving CDS practice. The topic guide was developed by authors L.R. and K.C., who have profound expertise in the literature on CDS and in conducting in-depth interviews. All interviews were conducted face-to-face and were held at the location chosen by the participants, which was always their professional practice. Interviews were audio-recorded using digital voice recording devices. A professional transcription service transcribed the interviews verbatim. 

### 2.6. Primary Study: Ethical Considerations

The Medical Ethics Committee of the Brussels University Hospital (2019/011) approved the primary study (B.U.N. 143201938601; 23 January 2019).

We assigned pseudonyms to all respondents in the transcripts and removed any identifying information. All physicians provided written informed consent to participate in the study.

### 2.7. Secondary Study: Secondary Qualitative Analysis

We performed a secondary qualitative analysis on the data from our primary study [23,25,26,27]. Given the research question of the secondary study, the overall process of our secondary analysis was informed by the theoretical framework of new ecology of social practice, which merges the ecological and constructivist traditions [43,44]. This implies that we considered CDS practice as a social practice consisting of interconnected and dynamic ecological systems or levels, namely, micro, meso, and macro levels [43,44,45,46]. Moreover, following the constructivist thinking within a new ecology of social practice, we aimed to capture the environmental context as the whole of multiple, subjective realities or factors rather than as one single, objective reality [43,44]. More specifically, our purpose was to identify these factors and situate them within ecological levels instead of merging them into one interplay in order to obtain ‘one environmental context’ of CDS practice. To that end, we considered the individual experiences and perceptions of physicians as the unit of analysis, which is in line with the primary data. 

All the in-depth interviews conducted for the primary study were included in the secondary qualitative analysis. This resulted in the analysis of the experiences and perceptions of 47 physicians. Full transcripts of the in-depth interviews were analyzed, namely, physicians’ answers to all the questions in the topic guide. To increase the rigor, we used uncoded transcripts for the secondary analysis to minimize the influence of the primary analysis [27].

The secondary analysis drew upon an interpretative thematic approach using inductive and deductive coding and theme development throughout different phases iteratively [30,47,48]. This approach is characterized by applying systematic procedures for identifying themes in the data that are common, dominant, or significant [30]. NVivo 12 software was used to organize the transcripts into structured codes and themes. 

The first phase of the secondary analysis included open coding and categorization of the raw data [30]. The purpose was to identify all factors and influences impacting physicians’ practices of CDS in the raw data. To begin with, authors S.V. and S.D. independently analyzed a first set of 12 interviews to identify codes in relation to such factors and influences considering the inherent meanings in the transcripts. Codes were created by attributing meaning to text fragments. S.V. and S.D. developed their own coding frames based on the coding of the 12 interviews. Thereafter, the coding frames were compared with each other. Differences and inconsistencies were discussed until consensus on one preliminary coding frame was reached. S.V. employed the merged coding frame to code the other transcripts. The coding frame was modified by means of constant comparison when new codes emerged. After the coding of all transcripts, S.V. and S.D. critically evaluated all codes. Consequently, some codes were grouped together, resulting in 42 codes.

The second phase involved the development of themes related to the context of CDS practice [30]. To that end, S.V. and S.D. merged the 42 codes into broader categories based on their similarities and relationships in a iterative and consensus-based process. In total, 22 themes were identified. Following the theoretical framework of a new ecology of social practice, they situated the identified themes onto one of the three ecological levels: micro, meso, and macro [43,44]. Micro was approached as the context of influence that originates from individuals and their interactions [49]. Meso was approached as the context of influence that is embedded in medium-level systems: institutions, organizations, settings, groups of individuals and populations, communities, etc. [49]. Macro was approached as the context of influence that is embedded in large systems: society, public policies, government, legislation, economy, societal fields (e.g., medical or educational), etc. [49]. This resulted in an initial framework of contextual factors impacting CDS practice.

The third phase included generating a final framework that encompassed the phenomenon being studied, i.e., the environmental context of CDS practice, in an iterative and consensus-based process including four group meetings with all coauthors. Firstly, we discussed the initial framework of contextual factors impacting CDS practice. We reassessed the meanings of all identified themes and to what extent they differed from each other. Secondly, we also reassessed whether they really belonged to the micro, meso, or macro level. After this ‘horizontal assessment’, we considered whether certain patterns were apparent ‘vertically’ across the themes. With regard to the meso and macro levels, i.e., the environmental context, we deemed that the identified themes could be clustered together around culture and structure. In this way, we also adhered to the new ecology of social practice in that research should pay attention to the notion of culture and structure, as both might steer individual behavior and social agency [43,44]. In accordance with this framework, we considered culture and structure as reciprocal spheres of influence [43]. In other words, structural factors were assumed to impact cultural factors and vice versa. The third phase resulted in a final framework of twelve environmental factors impacting physicians’ practices of CDS. Below, we discuss the content of these factors in detail. 

## 3. Results

### 3.1. Participant Characteristics

Forty-seven physicians were included in the secondary analysis (Table 1). The mean length of the in-depth interviews was 44 min. Participants had a professional background in oncology, general practice, intensive care medicine, geriatrics, and anesthetics. More than half of the participants had completed additional training in palliative medicine. Most participants were medical specialists in the hospital setting. The average age of participants was 46 years, and the majority were male. Seven out of ten participants had performed CDS at least six times in the 12 months prior to the interview.

### 3.2. Environmental Factors

**Figure 1:**
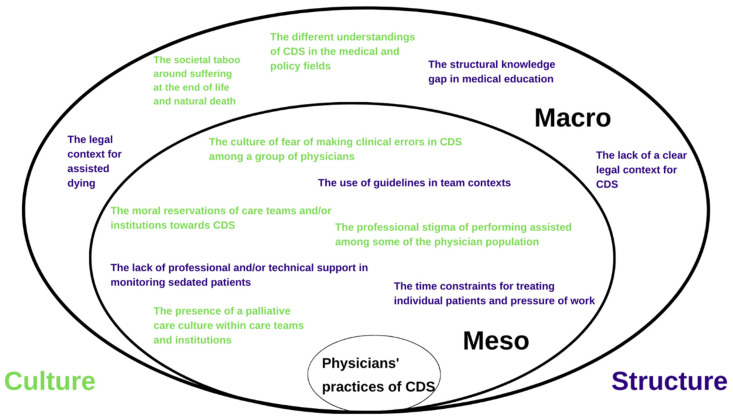
Physicians’ experiences and perceptions of the environmental factors affecting their practices of continuous deep sedation until death (CDS). The specific positions of the environmental factors identified at the meso and macro levels in the figure have no intrinsic meaning in terms of influence, but were chosen to make it clear that all factors are intertwined.

An overview of the environmental factors identified by our analysis can be found in Figure 1. The factors were categorized into cultural and structural, as we believed these overarching categories most accurately resonated with them. We defined structure as the entirety of systematically organized objects, parts, and elements. Culture, on the contrary, was defined as the entirety of shared views, attitudes, perceptions, norms, opinions, customs, and values. 

For analytical clarity, all environmental factors are presented in this article as distinctive features. However, it should be noted that, in reality, the factors are often intertwined. In the following sections, we describe the environmental factors in detail using participants’ spoken words (speech marks) and verbatim quotations (italics) to add depth and richness to the reporting of our findings [50].

#### 3.2.1. Structure: Meso

##### The Lack of Professional and/or Technical Support in Monitoring Sedated Patients

Participants hold that monitoring continuously deeply sedated patients is important, as it assesses the occurrence of awareness and unbearable suffering. To that end, they often rely on technical and/or professional support in settings in which they cannot carry out the monitoring themselves. In settings with limited or no professionals and/or infrastructure, participants often decide to induce CDS with a higher dose of sedatives than necessary to ensure that patients do not regain consciousness during CDS. Furthermore, some participants hesitate to initiate CDS when there is lack of support, or they postpone it. This is true in nursing homes and home settings in particular.

*“As I am the physician, I always initiate the first step. And that works well. But it is up to the nurse to actually put their chair next to bed, so to speak, and adjust the doses so that the patient doesn’t wake up. You have to act very quickly. So that implies that you need to be with the patient all the time. In a nursing home, CDS cannot go well. Mostly there is only one nurse practitioner responsible for an entire corridor with patients. Here, too, where there is a lack of nurse practitioners, I don’t dare to initiate CDS.”*

(Geriatrician)

##### The Use of Guidelines in Team Contexts

Among most participants working in a team context, such as a hospital, the use of guidelines has a “constraining” impact on CDS decision-making. Some participants say that their teams use existing or modified guidelines for CDS, for example, the guidelines for palliative sedation of The Federation of Palliative Care Flanders, to steer the practice of its team members. These guidelines stipulate in detail how the decision-making and performance of CDS “must” be done. Consequently, participants indicate that they sometimes adjust their CDS practice in accordance with these guidelines, even when the instructions in the guidelines are not appropriate for some cases. The constraining impact especially originates from their accountability to the team for deviating from the guidelines. In this regard, some participants point out that they have to report in detail to the team each time they do not adhere to the guidelines. 

##### The Time Constraints for Treating Individual Patients and Pressure of Work

Several participants, especially those with a background in general practice, indicate that “time and performance pressure” occasionally hinders them from carrying out CDS in the way they initially intended to. More specifically, the pressure sometimes “compels” these participants to initiate CDS with a high dose of sedatives to shorten the overall duration of CDS, since “time-consuming” monitoring after proportional initiation is impossible. They believe that government policy provoked this pressure of work: specifically, the health financing system requires physicians to treat “as many patients as possible in the shortest possible time”.

*“On the one hand, there is this extensive guideline. Firstly, it take some time to read it. Secondly, it devotes many pages to the conditions of CDS. This is explained in great detail. Then there is a short summary, which mainly focuses on the dosages in which starting schedule is added. The drugs have to be monitored every two hours. In practice, this is not always that easy. Monitoring CDS the way we are supposed to according to the guidelines. As a general practitioner, you lose a lot of time. You have to check the patient regularly during the CDS. That is not always possible in practical terms. Especially since they want us to treat more and more patients. So it is a matter of increasing the doses sometimes.”*

(General practitioner)

#### 3.2.2. Structure: Macro

##### The Structural Knowledge Gap in Medical Education

Nearly all participants claim that medical education does not focus on CDS. Furthermore, the lack of education has yielded a “structural knowledge gap” about CDS in the overall physician population. Therefore, they experience that this gap leads physicians to make “recurring errors” in terms of decision-making about and performance of CDS. Frequently cited errors include the use of suboptimal drugs and initiating CDS with the “incorrect intention”, namely, not for mitigating the unbearable suffering.

*“There is insufficient training for CDS and also insufficient knowledge. I have never had training on palliative sedation either. I never had anything about palliation. So a lot of doctors do not know what CDS means. Whatever their background is, they just think: “We will give them a bit of Midazolam and they will sleep. If it needs to go a bit faster? Then we should give them a bit more. After that, we add a little morphine. They think that CDS is pretty simple.”*

(Geriatrician)

##### The Legal Context for Assisted Dying

Several participants experience that a certain group of fellow physicians sometimes performs CDS to “intentionally” hasten the death of patients at the end of life instead of performing assisted dying in order to circumvent the procedural safeguards of assisted dying legislation, as these are perceived to be “complex” and “burdensome”. In addition, some participants report that they have, in exceptional situations, carried out CDS to hasten death in patients for whom a legal framework regarding end-of-life decisions was lacking, but only at the patients’ or their proxies’ repeated requests, such as in patients with a severe decline in mental capacity.

*“There are also a number of questions in that registration document about assisted dying that are not always easy to answer, for example, that you have to specify what exactly is unbearable suffering. That is one thing. And then there is the practical side of the registration. You have to take it to the post office. You have to send it by registered mail.. You have to register all of this in your patient file. In itself, that is quite a lot of work for something in which we have already invested a lot of time and energy. And I know that many general practitioners often think: “You know, I have really invested a lot of time, energy and effort for this, for which I have been paid little or nothing. But I also have to spend another half hour filling in the paperwork for the registration and another half hour going to the post office to get everything done, waiting in line and so on.” I also notice that colleagues ask questions about this and that this is a barrier preventing them from doing it. And then sometimes CDS is chosen.”*

(GP)

##### The Lack of a Clear Legal Context for CDS

Some participants argue that the “lack of a clear legal context for CDS” influences their decision-making and performance of CDS, as there is no legal basis for the elements it must include. Therefore, these participants perceive they have more room for interpretation regarding indications for CDS. Furthermore, some participants rely on the French legal context for CDS, i.e., the Claeys–Leonetti Law, to guide their CDS decisions. More specifically, they feel supported by that legislation to initiate CDS at patients’ explicit requests when most indications are fulfilled.

*“I think it is desirable for there to be legislation on CDS and for it to be written a bit like a guideline of how to carry things out, that you have to start at a certain dose depending on the weight of the patient, and that you then have to re-evaluate and document whether the patient is comfortable or not, and that you may then adjust the drugs proportionally. I think everyone has their own method now, because there is very little that is clear and black-and-white due to the lack of legislation.”*

(Intensive care physician)

#### 3.2.3. Culture: Meso

##### The Moral Reservations of Care Teams and/or Institutions towards CDS

Several participants explain that they often “hesitate” to perform CDS, as their care teams and/or institutions are reluctant to practice CDS. This reluctance partially stems from the moral reservations of these care teams and institutions towards CDS, considering it “morally pernicious”, as they believe that physicians mainly use it for terminating patient lives rather than for alleviating unbearable suffering. Given these moral reservations, some of these participants did not perform CDS in some cases, despite their belief of it being the best option for the patient, or they did not sedate the patient as deeply as they should have done.

*“The annoying thing is that you always get caught up in all those ethics. Some physicians say: “You cannot do that. Being so deeply sedated? And is that ethically acceptable?” So yes, in that case I try to find the middle ground based on what I know from all the fields. And searching, and gaining experience. For example, I consult the professionals from intensive care, to achieve something that is acceptable for me, but also for them. How should I put it? Intellectually acceptable. But yes, in the end, the patient is not deeply sedated.”*

(Palliative care physician)

##### The Presence of a Palliative Care Culture within Care Teams and Institutions

According to most participants, both those with training in palliative medicine as well as those without, the presence of a palliative care culture within their care team and/or institution facilitates their own performance of CDS. Palliative care culture is described by participants as the systematic use of palliative care by the care team and/or institution. Due to this palliative care culture, these participants feel encouraged to carry out CDS as palliation against refractory symptoms.

*“The biggest difference I see is in the nursing home where I am involved. I think we have a good palliative culture there, and we have also had a palliative care coordinator for the last year who was brought in solely for palliative care. We certainly give all the information about palliative care and CDS. So if you ask, “does everybody know what CDS entails?”, then it is certainly the intention for all our nurses to know what it means. When CDS is initiated, the coordinator will take the lead and support me and the nurses who sometimes take over the monitoring, but he will also support the resident’s family. That makes you feel, of course, as a GP, that you’re working in a medically authorized way and that makes things much easier.”*

(GP)

##### The Culture of Fear of Making Clinical Errors Regarding CDS among a Group of Physicians

Several participants experience a “culture of fear” present among a group of physicians surrounding clinical errors regarding CDS. More specifically, this group of physicians is fearful of administering CDS to patients who are not eligible for it in terms of not meeting all the required indications. The fear of these physicians stems from their common perception that CDS has “far-reaching” consequences for patients and their proxies, such as the inability to engage in social interaction. Because of this culture of fear, these physicians occasionally decide not to perform CDS. 

##### The Professional Stigma of Performing Assisted Dying among Some of the Physician Population

Several participants state that they have used CDS in part to end the lives of patients, rather than assisted dying, because of the professional stigma of performing assisted dying among some of the physician population. More specifically, this professional stigma means that performing assisted dying is viewed as physicians choosing the “easiest option” to end the suffering of patients. Because these participants do not wish to be associated with this, they sometimes use CDS in cases where they would otherwise use assisted dying.

*“And then this ‘compromise’. Yes, I think that this is often done. I have already experienced situations in which I discuss a case with medical specialists about a patient with cancer who had requested assisted dying and they say: “No, we really cannot do that. And if the suffering gets too severe, then we can always do CDS.” So then this compromise is chosen. There should be a chance to be able to talk about life-shortening actions without coming before this ‘moral court’.”*

(General practitioner)

#### 3.2.4. Culture: Macro

##### The Different Understandings of CDS in the Medical and Policy Fields

Some participants experience different understandings of CDS in the medical field that impact their practice. These understandings include the notion that CDS should be approached either as a standard practice or an exceptional one. According to these participants, the tension between these understandings in the medical field is reflected in a “jumble” of different indications, intentions, recommendations, and prudence surrounding CDS. Moreover, some participants report being hesitant and/or cautious to perform CDS, as it is viewed as an exceptional practice. Other participants, on the contrary, feel “empowered” to perform CDS, as it is a standard practice. According to the participants, this tension is also present in the field of policy. Here, they frequently refer to the ongoing policy debate about whether or not to implement control measures for CDS in Belgium.

##### The Societal Taboo around Suffering at the End of Life and Natural Death

Some participants often encounter pressure from patients and proxies to initiate CDS at the end of life. Participants attribute this pressure they experience to the societal taboo around suffering at the end of life, resulting in the fear of natural death and suffering in patients and their proxies. Because of this fear, they request CDS, as being sedated is deemed a “painless and peaceful way to die”. Consequently, participants indicate giving in to such pressure in certain cases, even though some patients do not fulfill the whole range of indications for CDS.

*“That sometimes CDS is initiated too early is also due to pressure from the families. “And look at him lying here now. That is not good, let him sleep now.” And then, as a physician, you hit a bit of a wall and sometimes the dose is increased abnormally or other things are done with the intention of speeding things up. But then that is not always what was initially intended. But it is very often under pressure from our society that these things happen. We are not always used to seeing people die. But maybe that is a reality that we have to learn to deal with as physicians? And, yes, dying, they want you to keep people alive for as long as possible. But you should not talk about it too much. You should not do too much advanced care planning. But when they lie there. You cannot do that. That is not possible. Yes, it has to be ‘done’ yesterday rather than today. And that is the current society in which we are living nowadays.”*

(Intensive care physician)

## 4. Discussion

### 4.1. Summary of Main Findings

We aimed to explore physicians’ experiences and perceptions of environmental factors affecting CDS practice. Multiple environmental factors were identified that could be centered around culture and structure. Firstly, structural factors include (1) the lack of professional and/or technical support in monitoring sedated patients; (2) the use of guidelines in team contexts; (3) the time constraints for treating individual patients and pressure of work; (4) the structural knowledge gap in medical education; (5) the legal context for assisted dying; and (6) the lack of a clear legal context for CDS. Secondly, cultural factors include (1) the moral reservations of care teams and/or institutions towards CDS; (2) the presence of a palliative care culture within care teams and institutions; (3) the culture of fear of making clinical errors regarding CDS among a group of physicians; (4) the professional stigma of performing assisted dying among some of the physician population; (5) the different understandings of CDS in the medical and policy fields; and (6) the societal taboo around suffering at the end of life and natural death. 

### 4.2. Strenghts and Limitations 

Since the influence of the environmental context on the practice of CDS has rarely been addressed by previous research, our secondary study represents an important contribution to the body of literature. All the researchers of the primary study were involved in the process of secondary analysis, and thereby were closer to the research subject, the study context, and the primary data [25,31,51]. By doing so, we enhanced the clarity and trustworthiness of the secondary study. For example, a misinterpretation of the methods used in the primary study was not possible [52]. Additionally, the primary data were collected recently. Therefore, it is most likely that the findings of our secondary analysis still apply to the current context of CDS practice [27,31]. The sample of participants shows a considerable degree of heterogeneity, which allowed us to include different perceptions and experiences in the analysis.

Performing a secondary study also entails some limitations. The use of a secondary design is a limitation in that the topic guide was not specifically geared to the research questions in this article. This may have led us to miss relevant information. Although theoretical sampling of participants was not possible to enhance the rigor of the secondary analysis, we argue that this limitation has been moderated by including a maximum variation of physicians’ experiences and perceptions in the analysis [29,31]. Although our ‘closeness’ to the primary study involves several opportunities, this simultaneously hinders a ‘tabula rasa’ approach in performing a secondary analysis [27]. Furthermore, environmental factors were identified with the aid of physicians’ experiences and perceptions. Therefore, we may not have captured the whole (inter)subjective complexity of the environmental context, as other parties involved were not included. In addition, we employed investigator triangulation, namely including multiple researchers in the whole process of analysis, to reduce potential bias. 

### 4.3. Interpretation of the Findings

In the general literature, the medical practice of physicians is assumed to be bound to an ever-changing and dynamic context, thus not merely being the results of individuals’ cognitive processes [53,54]. With regard to CDS practice, this assumption is also echoed by the narratives of participants in our study, indicating that the environmental context influences physicians’ practice of CDS. In this way, our findings could be interpreted as an empirical substantiation for the assumptions of several authors that the environmental context plays a non-negligible role in CDS. Although authors especially suggest the potential influence of culture [4,55], structure should not be overlooked to understand the full scope of factors influencing CDS practice, as multiple structural factors that have often been omitted by other research were identified in our study. By identifying environmental factors, our study might provide additional explanations for the considerable variation in prevalence and practice of CDS between settings and countries [5]. The latter, for instance, is reflected in the international UNBIASED Study conducted in the Netherlands, Belgium, and the United Kingdom [9,10]. That study showed that CDS could refer to a spectrum of practices, or variation in CDS practice, which was mainly attributed to the differences between practitioners themselves in these countries, such as differences in their personal attitudes towards CDS and conceptualizations of CDS [8]. Our findings suggest that such variations in CDS practice may not only be attributed to (inter)personal factors, but simultaneously to meso and macro factors. In that regard, some meso and macro factors identified could also be interpreted as facilitators that might prompt physicians’ use of CDS, namely, the presence of a palliative care culture within care teams and institutions, the shared understanding of CDS as a normal practice, assisted dying legislation, and societal fear of suffering at the end of life. Thus, hypothetically, these factors may partly explain why the prevalence of CDS in Belgium is considerably higher than in other countries [5].

Our study suggests that the practice of CDS is empirically intertwined with the practice of assisted dying, which corroborates previous studies [9,56,57,58,59,60,61,62]. More specifically, our findings indicate that in some cases, consideration of assisted dying is diverted to CDS due to the professional stigma of assisted dying among some of the physician population, the procedural requirements of the assisted dying legislation, and the gaps in this legislation in terms of excluding certain patient groups. This environmental context thus seems to influence CDS practice, leading to CDS being more often opted for as well as potentially being carried out with the intent of actively hastening death. In the literature, references have been made in that regard to ‘hidden assisted dying’, ‘slow assisted dying’, and ‘grey area practice’ [4,63]. Empirical studies, however, locate the causes of such intertwining to physicians in particular: for example in their moral identities [9] or their lack of an accurate notion of CDS and/or assisted dying [58]. Our findings, by contrast, suggest that the empirically close relationship between CDS and assisted dying is also determined through structural and cultural factors on the meso and macro level. In that regard, some participants in our study indicated the legal context for assisted dying in particular as a predominant macro factor of influence. This might contain important implications for those jurisdictions seeking to implement legislation on assisted dying. As such implementations can be accompanied with a change in how CDS is framed and approached both medically and societally, it is advisable for these jurisdictions to consider this potential change in their implementation process and to anticipate its effects.

### 4.4. Recommendations and Implications

As the practice of CDS is believed to have room for improvement, various quality improvement initiatives have been developed in the medical field that have had little impact [22]. In accordance with our findings, it is recommended that such initiatives should also address the environmental context of physicians in order to enhance their leverage. Guidelines for CDS, for instance, could also pay attention to how practitioners can deal with pressure from patients and their proxies to perform CDS, or to cope with their possible fear of making clinical errors in relation to CDS. Alternatively, guidelines for CDS that make a clear distinction between assisted dying and CDS may aid physicians to better differentiate between them in practice. 

The criticism of CDS has led public health policymakers in some countries, such as Belgium and the Netherlands, to consider how to improve CDS practice as a whole [63]. This study provides indications of which aspects to target. A first step in that direction might be that public health policymakers and the medical field work in concert to clarify the conceptual ambiguity regarding CDS. It also seems appropriate that they apply a contextual approach adapted to the real-life experiences of physicians to address the identified environmental factors that lead them to consider CDS as a substitute for assisted dying. Profound instruction about CDS in medical training is also a worthwhile recommendation. 

Future research could investigate to what extent our findings about the influence of the environmental context apply to countries without legislation on assisted dying. Another interesting research direction would be to investigate how and to what extent the implementation of cultural and structural elements in improvement initiatives would enhance CDS practice. Moreover, we only used the experiences and perceptions of physicians to explore environmental factors impacting CDS practice. Therefore, it may be interesting for future studies to explore the experiences of other involved parties, for example, nurses, to shed light on another part of CDS practice and to compare their accounts of environmental factors with our findings. Furthermore, further research is also needed on how micro factors in physicians’ practice of CDS are related to the meso and macro factors we have identified in this study. 

## 5. Conclusions

Based upon physicians’ experiences and perceptions, this study suggests that multiple environmental factors might affect their practice of CDS. This indicates that the environmental context should be considered to grasp the whole reality and scope of influence of CDS practice. The factors identified here could provide guidance to those who want to steer or intervene in physicians’ practice of CDS, underlining the need to move beyond physicians’ personal and interpersonal characteristics. Our findings emphasise the importance of adopting a whole-system approach to improve CDS practice as a whole.

## Figures and Tables

**Table 1:** Characteristics of the 47 participants included in the analysis.

Medical Specialty	*N* (%)
Oncology	13 (28)
General practice	13 (28)
Intensive care medicine	12 (26)
Geriatrics	8 (17)
Anesthetics	1 (2)
Additional medical training in palliative medicine *	25 (53)
Professional care setting	
Hospital	29 (62)
Home	18 (38)
Age	
<35 years	7 (15)
35–44 years	8 (17)
45–54 years	15 (32)
55–64 years	12 (26)
>64 years	5 (11)
Sex	
Male	26 (55)
Female	21 (45)
Number of patients treated who had died in the 12 months prior to the interview	
none	0 (0)
1–5 patients	2 (4)
6–10 patients	7 (15)
>10 patients	38 (81)
Number of continuous deep sedations performed in the 12 months prior to the interview	
none	0 (0)
1–5 patients	14 (30)
6–10 patients	5 (11)
>10 patients	28 (59)

* In Belgium, palliative medicine is a medical subspecialty for physicians provided as postgraduate training.

## Data Availability

Qualitative data in text format and pseudonymized databases will be stored in the archive of the Vrije Universiteit Brussel and will be shared with third parties only upon reasonable request and upon signing a data user agreement, as this data is covered by the GDPR (restricted access).
